# Asthma Prevalence in the Disaggregated Multiracial Population of California

**DOI:** 10.1001/jamanetworkopen.2024.49588

**Published:** 2024-12-09

**Authors:** Tracy Lam-Hine, Neeta Thakur, Aliya Saperstein, Mahasin S. Mujahid, David H. Rehkopf

**Affiliations:** 1 Department of Epidemiology and Population Health, Stanford University School of Medicine, Palo Alto, California; 2 Center for Population Health Sciences, Stanford University School of Medicine, Palo Alto, California; 3 Department of Medicine, University of California San Francisco School of Medicine; 4 Department of Sociology, Stanford University, Stanford, California; 5 Program in Human Biology, Stanford University, Stanford, California; 6 Division of Epidemiology, School of Public Health, University of California, Berkeley; 7 Department of Health Policy, Stanford University School of Medicine, Stanford, California; 8 Department of Medicine, Stanford University School of Medicine, Stanford, California; 9 Department of Pediatrics, Stanford University School of Medicine, Stanford, California

## Abstract

This cross-sectional study examines current and lifetime asthma prevalence among adults of all racial and ethnic categories in California, including the multiracial population.

## Introduction

Multiracial US adults have disproportionately high prevalence of current asthma (13%) and asthma attacks (55%) compared with other racial and ethnic groups.^1^ This poorly understood disparity could originate from stratifying effects of structural racism.^2^ Prior studies found some multiracial subgroups (eg, American Indian or Alaska Native–White, non-Hispanic Black-White) had higher lifetime asthma prevalence than monoracial counterparts.^3,4^ However, asthma surveillance generally does not disaggregate the multiracial category. Thus, other vulnerable subgroups’ experiences could be obscured by aggregation fallacy. This cross-sectional study assessed asthma disparities by disaggregating current and lifetime prevalence within all racial and ethnic categories, including multiracial.

## Methods

The Behavioral Risk Factor Surveillance System (BRFSS) conducts telephone surveys assessing adult prevalence of health-related factors. Detailed race and ethnicity data (all racial and ethnic groups selected) are currently not publicly available in the national BRFSS. Thus, we requested data from the California Department of Public Health, as California has the largest multiracial population of any state. We pooled all years with detailed race and ethnicity data (2014-2022) to maximize inclusion of smaller groups. We preserved self-identified races for multiracial participants and national origin for monoracial participants. We grouped Hispanic participants of any race. Following BRFSS reporting guidance, we reaggregated monoracial groups with fewer than 50 individuals into more general categories. For multiracial groups with fewer than 50 individuals, we reaggregated by prioritizing American Indian or Alaska Native and Native Hawaiian or Pacific Islander identification to avoid “data genocide” (systematic erasure of marginalized groups from population data).^5^ We stratified estimates by detailed race and ethnicity as a proxy for observing effects of structural racism and report crude weighted prevalence, as covariate adjustment would distort and understate real disparities. The Stanford University institutional review board exempted this study and waived informed consent because data were deidentified. The eMethods in Supplement 1 include additional details. We followed the STROBE reporting guideline.

## Results

Among 88 201 total participants (48.6% female, 47.3% male, 4.1% missing data), 35.2% identified as Hispanic (any race). Of non-Hispanic groups, 0.6%, were American Indian or Alaska Native; 15.4%, Asian; 5.5%, Black; 0.3%, Native Hawaiian or Pacific Islander; 39.0%, White; 2.0%, multiracial; and 1.9%, unknown race. Overall, 14.2% had been diagnosed and 8.4% still had asthma (Table).

**Table.** Participant Counts and Prevalence of Adult Current and Lifetime Asthma by Detailed Race and Ethnicity Categories From the California Behavioral Risk Factor Surveillance Survey, 2014-2022

Ethnicity, race	Participants, No. (weighted %)	Lifetime prevalence		Current prevalence	
		% (95% CI)^a^	RSE^b^	% (95% CI)^a^	RSE^b^
Hispanic					
Any race^c^	26 816 (35.2)	11.7 (11.1-12.4)	2.8	6.7 (6.2-7.2)	3.8
AIAN	1560 (2.0)	16.9 (14.0-20.4)	9.6	9.9 (7.7-12.7)	12.8
AIAN-White	146 (0.2)	16.7 (10.2-26.0)	23.7	10.1 (5.4-18.2)	31.0
Asian	259 (0.3)	23.2 (15.0-34.0)	20.9	15.0 (8.0-26.2)	30.3
Black	866 (1.3)	12.5 (9.0-17.1)	16.5	6.7 (3.9-11.1)	26.7
Black-White	63 (0.1)	21.0 (6.2-51.9)	54.9	18.9 (4.9-51.7)	61.3
Mexican	5014 (6.9)	10.5 (9.2-11.9)	6.7	5.5 (4.7-6.5)	8.5
Pacific Islander	98 (0.1)	20.6 (11.1-34.8)	28.9	19.0 (9.9-33.5)	31.0
Puerto Rican	90 (0.1)	7.0 (3.4-13.8)	35.4	4.4 (1.9-10.0)	42.3
White	17 091 (22.0)	11.4 (10.6-12.2)	3.5	6.7 (6.1-7.3)	4.8
Multiple^d^	243 (0.3)	8.6 (5.7-13.0)	21.2	3.9 (2.3-6.4)	26.0
Other	1386 (1.9)	11.3 (8.9-14.2)	11.9	4.7 (3.6-6.3)	14.4
Non-Hispanic monoracial					
AIAN	777 (0.6)	22.2 (17.9-27.1)	10.5	15.4 (11.8-19.8)	13.1
Asian					
All	7497 (15.4)	10.9 (9.7-12.1)	5.5	5.8 (4.9-6.9)	8.6
Asian Indian	1229 (2.1)	11.4 (8.8-14.5)	12.8	5.5 (3.7-8.0)	19.6
Chinese	1873 (3.6)	9.7 (7.4-12.5)	13.3	5.1 (3.2-8.0)	23.2
Filipino	1292 (2.6)	18.9 (15.5-22.8)	9.9	11.3 (8.4-15.1)	14.9
Japanese	379 (0.8)	9.9 (6.3-15.2)	22.4	5.9 (3.1-11.1)	32.8
Korean	387 (0.7)	7.3 (4.4-11.9)	25.2	2.6 (1.1-6.1)	43.7
Vietnamese	479 (1.0)	11.0 (7.8-15.4)	17.2	5.5 (3.5-8.4)	22.1
Other	957 (1.8)	5.6 (4.1-7.7)	16.3	2.9 (1.9-4.3)	21.0
Unspecified^e^	901 (2.8)	8.1 (5.7-11.5)	18.1	4.2 (2.5-6.8)	25.2
Black	4507 (5.5)	20.9 (18.8-23.1)	5.2	13.2 (11.6-15.0)	6.6
NHPI					
All	303 (0.3)	13.0 (9.1-18.1)	17.5	7.1 (4.3-11.5)	24.8
Pacific Islander	127 (0.1)	14.4 (8.7-23.0)	24.8	9.4 (4.9-17.2)	32.0
Samoan	50 (0.1)	13.9 (5.9-29.3)	40.1	8.0 (2.3-24.5)	59.5
Other Pacific Islander	126 (0.1)	11.1 (6.0-19.8)	30.5	4.4 (1.6-11.7)	51.1
White	43 595 (39.0)	16.3 (15.7-16.9)	1.9	10.0 (9.5-10.5)	2.6
Unknown race^f^	2418 (1.9)	14.6 (12.6-16.9)	7.5	7.6 (6.2-9.3)	10.6
Non-Hispanic multiracial					
All	2288 (2.0)	24.0 (21.3-27.1)	6.2	14.0 (11.8-16.7)	9.0
AIAN-Black	110 (0.1)	30.4 (20.6-42.3)	18.2	21.9 (13.4-33.8)	23.4
AIAN-Black-White	109 (0.1)	24.3 (11.9-43.2)	32.9	16.4 (5.9-38.0)	47.7
AIAN-White	590 (0.5)	22.4 (17.3-28.5)	12.7	14.1 (9.9-19.7)	17.6
AIAN-multiple	183 (0.1)	33.5 (23.1-45.8)	17.5	29.5 (19.3-42.2)	19.9
Asian-Black	62 (0.1)	18.8 (9.4-34.1)	32.3	5.0 (2.1-11.6)	43.0
Asian–Pacific Islander	112 (0.1)	21.7 (11.8-36.4)	28.6	14.1 (5.9-30.1)	41.7
Asian-White	456 (0.3)	18.9 (14.2-24.7)	14.1	6.6 (4.4-9.6)	19.7
Asian-multiple	75 (0.1)	9.4 (4.3-19.2)	37.5	4.1 (1.4-11.8)	54.5
Black-White	268 (0.2)	32.3 (23.3-42.7)	15.4	18.9 (12.1-28.2)	21.6
Black-multiple	77 (0.1)	24.7 (12.1-43.8)	32.7	7.7 (3.0-18.3)	45.8
Pacific Islander–White	58 (0.1)	20.6 (10.2-37.2)	32.6	7.2 (2.6-18.2)	48.4
Pacific Islander–multiple	68 (0.1)	33.0 (17.9-52.7)	27.2	11.7 (4.8-25.9)	42.4
Other race–White	120 (0.1)	23.1 (11.5-41.0)	32.6	17.1 (6.7-37.4)	44.2
Total sample	88 201 (100)	14.2 (13.8-14.6)	1.4	8.4 (8.1-8.7)	2.0

Abbreviations: AIAN, American Indian or Alaska Native; NHPI, Native Hawaiian or Pacific Islander; RSE, relative standard error.

^a^ Weighted crude prevalence. Wide 95% CIs indicate imprecision in prevalence estimates for smaller groups.

^b^ Larger RSEs indicate unstable estimates and should be interpreted cautiously.

^c^ Participants selecting a Hispanic subgroup but choosing “other” or “don’t know” or declining to identify a race were classified as the Hispanic subgroup they selected.

^d^ Groups with fewer than 50 participants selecting multiple Hispanic subgroups were classified as multiple Hispanic.

^e^ Participants selecting a race that allowed for more specific subgroup identification but who did not choose a subgroup were classified as unspecified.

^f^ Unknown race includes participants who did not provide informative responses to either race or Hispanic ethnicity questions (eg, “don’t know,” “other,” or declined to answer).

Lifetime and current prevalence was highest among American Indian or Alaska Native–Black (30.4% [95% CI, 20.6%-42.3%]; 21.9% [95% CI, 13.4%-33.8%]), American Indian or Alaska Native–multiple (33.5% [95% CI, 23.1%-45.8%]; 29.5% [95% CI, 19.3%-42.2%]), and Black-White (32.3% [95% CI, 23.3%-42.7%]; 18.9% [95% CI, 12.1%-28.2%]) adults and substantially higher than in the monoracial populations with which they shared identities (Table). The Hispanic subgroups with highest prevalence were Asian-Hispanic (23.2% [95% CI, 15.0%-34.0%]; 15.0% [95% CI, 8.0%-26.2%]), Black-Hispanic-White (21.0% [95% CI, 6.2%-51.9%]; 18.9% [95% CI, 4.9%-51.7%]), and Hispanic–Pacific Islander (20.6% [95% CI, 11.1%-34.8%]; 19.0% [95% CI, 9.9%-33.5%]). Stratification in prevalence was more pronounced for lifetime vs current asthma. Current prevalence was higher in these multiracial and Hispanic subgroups. Prevalence for both outcomes was lowest for Korean (7.3% [95% CI, 4.4%-11.9%]; 2.6% [95% CI, 1.1%-6.1%]), Other Asian (5.6% [95% CI, 4.1%-7.7%]; 2.9% [95% CI, 1.9%-4.3%]), and Puerto Rican (7.0% [95% CI, 3.4%-13.8%]; 4.4% [95% CI, 1.9%-10.0%]) adults.

## Discussion

This study extended prior asthma disaggregation studies^3,4^ to highlight previously unreported vulnerable populations. American Indian or Alaska Native–Black and American Indian or Alaska Native–multiple adults in California had disproportionate asthma burden, potentially reflecting intersecting marginalization based on monoracial and multiracial identities or other unmeasured risk factors specific to these groups. Asthma prevalence among Black-White multiracial adults was similar to previous studies^1,3^ but markedly lower among Puerto Rican individuals. Aggregation fallacies prevent effective surveillance of asthma disparities, which have improved little despite coordinated federal actions. We did not address bias from potential associations between racial or ethnic identity, asthma diagnosis, and survey nonresponse, a limitation. Investigators should examine asthma determinants among vulnerable multiracial and easily missed populations to maximize health data equity given these groups’ rapid growth.^6^

## Notes

Supplement 1.
eMethods.

Supplement 2.
Data Sharing Statement
