# Supplementary material for: Asthma Prevalence in the Disaggregated Multiracial Population of California
Source: JAMA Netw Open. 2024 Dec 9;7(12):e2449588. doi: 10.1001/jamanetworkopen.2024.49588 (PMC11629126; doi:10.1001/jamanetworkopen.2024.49588)
Supplement: Supplement 1. — eMethods. [file jamanetwopen-e2449588-s001.pdf]

Supplementary Online Content

Lam-Hine T, Thakur N, Saperstein A, Mujahid M, Rehkopf DH. Asthma prevalence in the disaggregated multiracial population of California. *JAMA Netw Open*. 2024;7(12):e2449588. doi:10.1001/jamanetworkopen.2024.49588

eMethods

This supplementary material has been provided by the authors to give readers additional information about their work.

© 2024 Lam-Hine T et al. *JAMA Network Open*.

Methods Supplement
Lam-Hine et al. in *JAMA Network Open*, 2024

Additional methodological details to support the STROBE checklist are described below:

- **Study design**: This study employed a cross-sectional design to estimate crude period prevalence (2014-2022) of asthma in California by detailed race/ethnicity.
- **Setting**: The Behavioral Risk Factor Surveillance System (BRFSS) is a cross-sectional survey of adults ages 18 and over administered annually by US state and territorial health departments with support from the Centers for Disease Control and Prevention (CDC). BRFSS collects information on health-related determinants, behaviors, chronic conditions, and access to care through interviews on landline telephones and cell phones. Jurisdictions use probability-based sampling for participant selection. Participant recruitment into BRFSS is on-going, and there is no longitudinal participant follow-up. Additional information about the BRFSS design is available at: https://www.cdc.gov/brfss/annual_data/2023/pdf/Overview_2023-508.pdf
- **Participants**: Eligible participants are non-institutionalized adults (18 and over) residing in the US and participating US territories.
- **Informed consent**: Informed consent was obtained verbally from all participants included in the BRFSS.
- **Variables**: The variables used in this analysis were (1) lifetime asthma diagnosis, (2) current asthma diagnosis, and (3) self-identified race/ethnicity categorized by the investigator.
- **Data sources/measurement**:
    - Lifetime asthma diagnosis: Interviewers asked participants, "Has a doctor, nurse, or other health professional ever told you that you had asthma? Please tell me yes, no, or you're not sure."
    - Current asthma: For participants responding "yes" to the previous question, interviewers then asked "Do you still have asthma? Please tell me yes, no, or you're not sure."
    - Ethnicity: Interviewers asked participants, "Are you Hispanic, Latino/a, or Spanish origin?" For participants indicating yes, interviewers asked "Are you Mexican, Mexican American, Chicano/a; Puerto Rican; Cuban; or "Another Hispanic, Latino/a, or Spanish origin?" Participants were allowed to select multiple responses, say they didn't know, or refuse to respond.
    - Race: After asking about Hispanic ethnicity, interviewers asked participants, "Which one or more of the following would you say is your race? White, Black or African American, American Indian or Alaska Native, Asian, or Pacific Islander?" For participants selecting Asian or Pacific Islander, interviewers provided Asian (Asian Indian, Chinese, Filipino, Japanese, Korean, Vietnamese, or Other Asian) and Pacific Islander (Native Hawaiian, Guamanian or Chamorro, Samoan, or Other Pacific Islander) national origin options as well. Participants were allowed to select multiple races, say they didn't know, or refuse to respond. There is no additional information provided in BRFSS documentation about respondents choosing any of these categories.
- **Bias**: Previous research (Schneider et al. 2012, BRFSS Summary Quality Data Report 2018) has shown that BRFSS response rates are lower than other population surveys, and that minoritized racial/ethnic groups are likely underrepresented in BRFSS even after accounting for non-response adjustment weights. These patterns suggest that there could be uncontrolled sample selection bias affecting the representativeness and accuracy of estimates derived from BRFSS data. Other than applying the BRFSS-calculated (landline and cellular) complex sampling weights, we did not attempt to address any other sources of bias (e.g.: information or selection bias) in the underlying datasets provided. This is in part due to the fact that there are no agencies that collect or report population estimates for the specific population subgroups studied in this analysis. Potential

© 2024 Lam-Hine T et al. *JAMA Network Open*.

associations between identification in these groups, asthma diagnosis, and nonresponse are a limitation of this analysis.

- **Study size**: After pooling the nine years (2014-2022) of California BRFSS data where detailed race/ethnicity data were collected, there were in total 88,201 participants. Participants providing uninformative responses to questions about lifetime or current asthma or were truly missing data on race/ethnicity were excluded from analyses of those outcomes (see below).
- **Quantitative variables**: Below, we describe the construction of the variables from the data source used in this analysis.
  - Lifetime asthma: We coded participants responding "yes" to the question about ever being diagnosed with asthma by a medical provider as having lifetime asthma. Participants responding "no" to the question were categorized as not having asthma. There were 277 (0.3%) participants that provided an uninformative response (don't know, refused, or missing). These participants were excluded from the lifetime asthma analysis.
  - Current asthma: We coded participants responding "yes" to the follow-up question about still having asthma as having current asthma. Participants responding "no" to the follow-up question were categorized as not having current asthma. There were 600 (0.6%) participants that provided an uninformative response (don't know, refused, or missing). These participants were excluded from the current asthma analysis.
  - Detailed race/ethnicity
    - To create our detailed race/ethnicity variable, we preserved all self-identified races for Multiracial participants, and national origin for monoracial participants. We did not preserve national origin selections for Multiracial participants due to sample size limitations among the overwhelming number of categories that would emerge.
      - Example 1: a participant who selected Hispanic (Mexican), American Indian, and White was coded as AIAN-Hispanic-White and counted in the Hispanic category
      - Example 2: a person who selected Asian (Vietnamese) was coded as Vietnamese
      - Example 3: a person who selected Hispanic (Mexican and Puerto Rican) was coded as Multiple Hispanic
    - Because BRFSS guidance recommends not reporting estimates for groups with N<50 participants, we re-aggregated detailed race/ethnicity subgroups with N<50 into larger groups based on the BRFSS race/ethnicity hierarchy structure.
      - Example 1: There were fewer than 50 people identifying as Cuban alone. Thus, we re-aggregated all Cubans into the "Other Hispanic" category
      - Example 2: There were fewer than 50 people identifying as Native Hawaiian alone. Thus, we re-aggregated all Native Hawaiians into a more general "Pacific Islander" category
    - For Multiracial subgroups where N<50, we could conceivably regroup Multiracial participants by any one of their racial identifications. However, previous literature (see Quint, Matagi, and Kaholokula 2023) has described the negative sociopolitical impacts of "data genocide", which includes categorizing indigenous (specifically, American Indian or Alaska Native, Native Hawaiian, or Pacific Islander) Multiracial people into an aggregate Multiracial category. This practice obscures health inequities that are rooted in colonialism, and erases indigenous life and health experiences. In order to promote health data equity (see Ponce, Shimkhada, and Adkins-Jackson 2023, Lam-Hine et al. 2024), we reaggregated prioritizing identification as American Indian or Alaska Native

© 2024 Lam-Hine T et al. *JAMA Network Open*.

(AIAN) or Pacific Islander. These new subgroups, “AIAN-Multiple” and “Pacific Islander-Multiple” combined Multiracial participants that identified as AIAN or as Pacific Islander but were previously in subgroups with N<50 participants. Secondarily, we prioritized groups based on prevalence of asthma among monoracial counterparts to preserve visibilities. The algorithm prioritized identification in the following order: (1) AIAN, (2) Pacific Islander, (3) Black, and (4) Hispanic and (5) Asian identification.

    - Example 1: There were fewer than 50 people identifying as AIAN-Black-Hispanic-White or as AIAN-Asian-NHPI. Thus, we re-aggregated participants from these two groups together, prioritizing AIAN identification, into a more general “AIAN-Multiple” category.

  - We grouped participants that responded in an uninformative way to the race question (Other, Don’t Know, Refused; N=1,178) or were truly missing race/ethnicity data (N=3) together in a general “Unknown” category, unless they indicated Hispanic ethnicity. In that case, they were categorized as Hispanic or the appropriate Hispanic subgroup.

- **Statistical methods**:
  - Covariate adjustment: Because the goal of this paper is to describe real disparities (not a counterfactual state), we did not control for covariates in this analysis. We did not adjust for age as is common in descriptive analyses, as there are no reliable population or age distribution estimates for the very specific subgroups presented in this paper, making traditional direct/indirect standardization approaches impractical.
  - Subgroup analyses: Our paper reports estimates stratified by detailed race/ethnicity. Despite known sex-based differences in asthma, we also chose not to stratify estimates by sex because the main goal of this paper was to maximize reporting of smaller, frequently overlooked groups, and further stratification by sex would render estimates even more unstable. This is a limitation of the data used in this analysis.
  - Missing data: We did not attempt to impute race/ethnicity for those missing or providing uninformative responses using common race imputation approaches such as Bayesian Information (First) Surname Geocoding, as we did not have access to participant names or geolocations and because these methods perform very poorly for Multiracial and AIAN populations (Elliot et al. 2009, Grundmeier, et al. 2015). We also did not attempt to impute missing asthma data, as none of the detailed race/ethnicity subgroups had more than 2.0% of respondents missing data for lifetime asthma and 5.9% for current asthma.
  - Sensitivity analyses: Research letters do not allow presentation of results from sensitivity analyses.

© 2024 Lam-Hine T et al. *JAMA Network Open*.
