# Supplementary material for: Asthma Prevalence in the Disaggregated Multiracial Population of California
Source: JAMA Netw Open. 2024 Dec 9;7(12):e2449588. doi: 10.1001/jamanetworkopen.2024.49588 (PMC11629126; doi:10.1001/jamanetworkopen.2024.49588)
Supplement: Supplement 2. — Data Sharing Statement [file jamanetwopen-e2449588-s002.pdf]

Data Sharing Statement

Lam-Hine. Asthma Prevalence in the Disaggregated Multiracial Population of California. *JAMA Netw Open*. Published December 09, 2024. doi:10.1001/jamanetworkopen.2024.49588

Data
Data available: No

Additional Information
Explanation for why data not available: Data are available upon request from the California Department of Public Health.
